# Lost in transition: Maize BAM7 is a dual function gene encoding a nuclear BAM7 and plastidial BAM2

**DOI:** 10.1093/plphys/kiad260

**Published:** 2023-05-04

**Authors:** Jiawen Chen

**Affiliations:** Assistant Features Editor, Plant Physiology, American Society of Plant Biologists, USA; John Innes Centre, Norwich Research Park, Norwich, NR4 7UH, UK

The evolutionary history of genes is shaped by gene fusions, duplications, and subfunctionalizations, introducing genetic diversity over time. The β-amylase (BAM) gene family in angiosperms presents an interesting example where 2 ancestral BAM genes are thought to have undergone many duplications and developed over different evolutionary pathways to result in 8 clades with 10 members that have distinct functions and subcellular localizations (Monroe and Storm 2018; Thalmann et al. 2019). This presents a complicated picture, where the exact phylogenetic history of the BAM family is not clear yet.

BAMs are starch hydrolases that are important for the mobilization of sugar reserves from starch. They reside in plastids and cleave α-1,4 glycosidic bonds at the nonreducing end of glucans, releasing maltose, which can be transported to the cytosol. BAMs are named after the β-glucose on the reducing end of the released maltose.

The best-studied BAMs are in *Arabidopsis thaliana* (Arabidopsis) chloroplasts, but even in Arabidopsis, the physiological function of some BAM genes is not yet clear, and some BAM-like genes no longer encode active enzymes or have different localizations. The plastidial AtBAM1, −2, −3, and −6 have a hydrolytic role in starch degradation, and AtBAM5 is catalytically active and cytosolic, whereas AtBAM7 and −8 are catalytically inactive transcription factors in the nucleus, and AtBAM4 and −9 are catalytically inactive and plastidial. The recently identified BAM10 is absent in Arabidopsis but found in other angiosperms and is likely also catalytically inactive and plastidial (Thalmann et al. 2019).

The exact evolutionary history of the functionally diverse BAM gene family is unclear, highlighted by the relationship between BAM2 and BAM7. BAM2 and BAM7 are closely phylogenetically related and are found in the same clade (Thalmann et al. 2019; Ravenburg et al. 2022) despite their different functions in Arabidopsis. AtBAM2 is a plastidial, catalytically active BAM. Although the physiological role of AtBAM2 is not known (Monroe 2020), biochemical studies have shown that it is a unique tetrameric BAM (Chandrasekharan et al. 2020). By contrast, AtBAM7 has very low BAM activity and is a nuclear transcription factor that influences shoot development. AtBAM7 contains a BRASSINAZOLE RESISTANT1 (BZR1)-like DNA-binding domain, which is also found in transcription factors that regulate brassinosteroid responses (Reinhold et al. 2011). The BZR-like domain binds a sequence that resembles a BR-responsive element, and *BAM7* genes likely originate from a fusion event of an ancestral *BAM2* with a BZR-like domain (Thalmann et al. 2019). Although the AtBAM7 BAM domain has very low activity, it is possible that it binds another ligand and integrates metabolic and brassinosteroid signaling (Reinhold et al. 2011).

The *Zea mays* (maize) BAM7 is interesting because it has all the residues needed for both a functional nuclear-localized BAM7 and a catalytically active plastidial BAM2, and maize does not contain any *BAM2* gene (Ravenburg et al. 2022). When expressed in *Escherichia coli*, the BAM domain of ZmBAM7 behaves like AtBAM2, forming a tetramer with sigmoidal kinetics. ZmBAM7 localizes to the nucleus when expressed in onion cells (Yu et al. 2018), but the physiological function of ZmBAM7 has not been studied.

**Figure 1.**
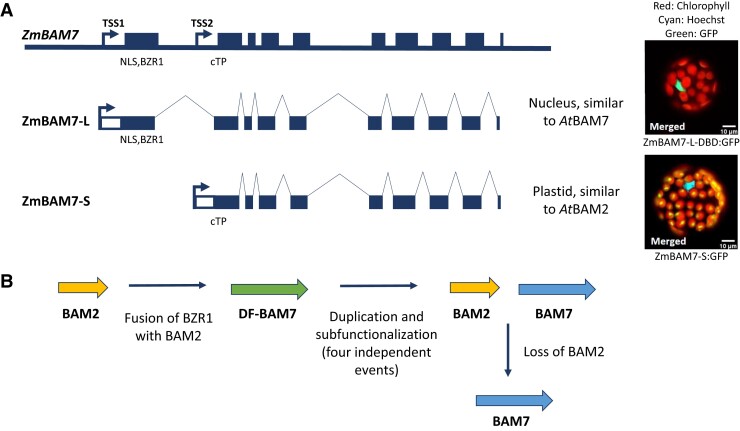
*ZmBAM7* encodes 2 transcripts that resemble *AtBAM7* and *AtBAM2.***A**) Gene models of the full-length *ZmBAM7* gene and the 2 transcripts generated from it. Exons (dark bars), introns (dark lines), and 2 putative TSSs are indicated. The 2 TSSs are approximately 1 kb apart. The approximate positions of the nuclear localization signal and BZR1-like DNA binding domain are indicated in the first exon, and the position of the predicted chloroplast transit peptide (cTP) is indicated in the second intron. White boxes are 5′ UTRs. On the right, the localization of the *Zm*BAM7-L DNA binding domain and the *Zm*BAM7-S protein expressed in Arabidopsis protoplasts are shown. Hoechst dye stains DNA and marks the site of the nucleus. This image was adapted from Ozcan and Monroe (2023). **B**) Schematic summarizing the events proposed during the evolution of BAM2 and BAM7, with *BAM2* as the ancestral gene. DF-BAM7 is the dual function BAM7 as found in *Z. mays*. To the right, the separate *BAM2* and *BAM7* genes are as they appear in Arabidopsis after duplication and subfunctionalization. Some species have also lost *BAM2* and retain only a specialized *BAM7*.

In this issue of *Plant Physiology*, Ozcan and Monroe (2023) elaborated on their previous work on the biochemical characterization of ZmBAM7 (Ravenburg et al. 2022) by performing more detailed analyses on the genetics and evolution of the *ZmBAM7* gene structure. *ZmBAM7* is a dual function BAM7 gene (DF-BAM7). The full-length gene contains 10 exons, with the first exon containing a BZR1-like domain and a putative nuclear localization signal (Fig. 1A). *ZmBAM7* expression was upregulated by abscisic acid (Yu et al. 2018) and ZmBAM7 is hypothesized to be a transcription factor due to its similarity with AtBAM7, but its exact function in the nucleus is not yet known.

The authors show that *ZmBAM7* has 2 alternative transcription start sites (TSSs) that encode a long transcript (*ZmBAM7-L*) resembling a canonical BAM7 and a shorter transcript (*ZmBAM7-S*) resembling a canonical BAM2. *ZmBAM7*-S has a TSS that starts in intron 1 of the full-length gene and contains a cryptic chloroplast transit peptide that is not present in *ZmBAM7-L* (Fig. 1A). The 2 transcripts were experimentally confirmed using 5′ Rapid amplification of cDNA ends, using cDNA generated from maize leaf tissue. Several bioinformatic analyses were conducted to provide further evidence for the 2 transcripts using publicly available data. Expressed sequence tag, full-length cDNA, and RNA-sequencing data all confirmed 2 sequences corresponding to the 2 unique transcripts. RNA-sequencing also revealed that the 2 transcripts are expressed in different abundance, where the relative abundances could vary depending on the tissue; this suggests independent regulation of *ZmBAM7-L* and *ZmBAM7-S*. In protoplasts of transgenic Arabidopsis lines expressing the ZmBAM7-L DNA-binding domain (DBD) or the full-length ZmBAM7-S with fluorescent tags, ZmBAM7-L-DBD localized to the nucleus and ZmBAM7-S to the chloroplast (Fig. 1A).

To understand the evolutionary history of *ZmBAM7* and other *DF-BAM7*s, the authors performed alignments and phylogenetic analyses. They aligned the amino acid sequences of BAM2 and BAM7 from 14 species that contain separate *BAM2* and *BAM7* genes and from 15 species that have *DF-BAM7* genes, demonstrating the prevalence of the dual-function gene structure. The authors also aligned amino acid sequences of the BZR1-like domain of BAM7 and the BAM domain of BAM7 and BAM2 from 61 species. From these alignments, they created phylogenetic trees that demonstrated that *BAM2* likely fused with a BZR-like gene to make *BAM7*, creating the *DF-BAM7* genes (Fig. 1B). Four independent duplication and subfunctionalization events then created separate *BAM2* and *BAM7* genes: in pteridophytes and gymnosperms and twice in angiosperms. Most eudicots also have the *BAM2* gene immediately upstream of *BAM7*, suggesting a tandem duplication. Interestingly, Solanaceae, Cucurbitales, and Fabales have subsequently lost *BAM2* and only have a *BAM7* (Fig. 1B).

These findings present *ZmBAM7* and other *DF-BAM7* genes as transitional states in the evolution of *BAM7* and *BAM2*. Although ZmBAM7-L is not likely to have a catalytic function in the nucleus, where there is no starch, further investigation is needed to confirm whether the intact catalytic domain has any additional function in the nucleus or might be binding to another ligand. More broadly, the physiological role for both ZmBAM7-L and ZmBAM7-S is not known, and further experiments such as mutant studies could provide more insight. Previous identification of alternative TSSs in plants was shown to have a function in dynamic light-regulated protein localization in phytochrome light sensing (Ushijima et al. 2017), but the current study describes 2 likely functionally unrelated proteins expressed from the same gene. Future studies should investigate whether the BAM alternative TSSs have any function in environmental responses and whether there is any metabolic crosstalk resulting from the dual function gene structure.
